# Hereditary thrombophilia

**DOI:** 10.1186/1477-9560-4-15

**Published:** 2006-09-12

**Authors:** Salwa Khan, Joseph D Dickerman

**Affiliations:** 1 Department of Pediatrics, University of Maryland, Suite N5W56 22 S. Greene St. Baltimore, MD 21201, USA; 2 Department of Pediatrics, University of Vermont College of Medicine, D201D Given Building 89 Beaumont Avenue Burlington, Vermont 05405, USA

## Abstract

Thrombophilia can be defined as a predisposition to form clots inappropriately. Thrombotic events during infancy and childhood are increasingly recognized as a significant source of mortality and morbidity. The predisposition to form clots can arise from genetic factors, acquired changes in the clotting mechanism, or, more commonly, an interaction between genetic and acquired factors. Since the turn of the last century, there has been extensive research focusing on both the genetic and acquired causes of thrombophilia, with particular focus on clotting events in the venous circulation. This review describes clinically relevant aspects of genetic venous thrombophilia, which include well-established, lesser known, and suggested causes of inherited thrombophilias.

## Background

Thrombophilia can be defined as a predisposition to form clots inappropriately. Thrombotic events are increasingly recognized as a significant source of mortality and morbidity [1]. The predisposition to form clots can arise from genetic factors, acquired changes in the clotting mechanism, or, more commonly, an interaction between genetic and acquired factors [2]. A hereditary thrombophilia results when an inherited factor, such as antithrombin or protein C deficiency, requires interaction with components that are inherited or acquired before onset of a clinical disorder [3]. A homozygous abnormality or combination of two or more heterozygous abnormal factors can lead to clinically apparent thrombotic disorders at an early age. However, milder heterozygous traits, when existing alone, are more often discovered by laboratory investigation. [3]. Since the turn of the last century, there has been extensive research focusing on both the genetic and acquired causes of thrombophilia, with particular focus on clotting events in the venous circulation. This paper will focus on clinically relevant aspects of genetic venous thrombophilia. While there is evidence for adverse outcomes of pregnancy associated with thrombophilia, an in-depth discussion of that area is beyond the scope of this article.

## Review of clotting and fibrinolysis

In 1856, Rudolf Virchow proposed a hypothesis to explain the etiology of pulmonary emboli, which lead to the understanding of the three primary causes of venous and arterial thrombosis: stasis, injury to the vessel wall and abnormalities in the circulating blood. Subsequently, numerous investigators elucidated the concept of a hemostatic balance between fibrin formation and fibrin dissolution.

As our insight into the hemostatic and fibrinolytic pathways has developed it has become apparent that there are specific factors that can create an imbalance in the clotting process and thus lead to thrombosis as originally suggested by Virchow. An understanding of both the coagulation and fibrinolysis pathway has helped to determine specific factors that can cause such an imbalance in hemostasis.

## Coagulation cascade

The coagulation and fibrinolytic systems are two separate but linked enzyme cascades that regulate the formation and breakdown of fibrin. The blood clotting system or coagulation pathway, like the complement system, is a proteolytic cascade. Each enzyme of the pathway is present in the plasma as a zymogen (an inactive form), which, on activation, undergoes proteolytic cleavage to release the active factor from the precursor molecule. The coagulation pathway functions as a series of positive and negative feedback loops which control the activation process. The ultimate goal of the pathway is to produce thrombin, which can then convert soluble fibrinogen into fibrin, which forms a clot. The generation of thrombin can be divided into three phases: the intrinsic and extrinsic pathways that provide alternative routes for the generation of factor X, and the final common pathway which results in thrombin formation.

Coagulation is initiated when factor VIIa binds to tissue-factor (TF) on the surface of endothelial cells and monocytes at sites of vascular injury. The TF-factor VII complex activates factor IX and X to factors IXa and Xa, respectively. Factor Va and Xa, together, activate prothrombin to thrombin. Thrombin has multiple prothombotic roles: it cleaves soluble fibrinogen to insoluble fibrin that will eventually form the hemostatic plug, and activates factors V, VIII, XI and XIII. Thrombin also acts to produce an anticoagulant effect by forming an enzyme complex with thrombomodulin to activate protein C. The tissue factor-VIIa complex is rapidly inactivated by the TF pathway inhibitor.

## Natural inhibitors of coagulation

Activated coagulation factors are serine proteases, and their activity is modulated by several naturally occurring plasma inhibitors. The most important inhibitors of the blood coagulation system are antithrombin, protein C, and protein S [4]. An inherited deficiency of one of these three proteins is found in about 15% of patients who present with venous thrombosis before the age of 45 [5].

Antithrombin (AT) is a serine proteinase inhibitor that plays a significant role in the process of coagulation by interaction with its co-factor, heparin. Antithrombin inactivates thrombin directly, and also inactivates factors IX, X and XI by forming a covalent complex. Inhibition of most of the factors is slow; however, the process can be accelerated at least 1000 fold by the binding of heparin and heparin-like compounds to AT [6]. Protein C is activated by thrombin, a process greatly enhanced by the interaction of thrombin with thrombomodulin. Activated protein C proteolytically inactivates factors Va and VIIIa on the platelet and endothelial cell surface and hence serves to block thrombin generation and the subsequent steps in coagulation. Protein C requires protein S, another vitamin K dependent molecule as a co-factor. The imbalance between reduced inhibitors of coagulation and/or increased activation of coagulation factors lead to thrombosis [4].

## Fibrinolysis

Specific enzymes are involved in the removal of blood clots from the circulation and the turnover of extracellular matrix proteins. One of the most important enzymes in this setting is plasmin. The main role of plasmin is to degrade fibrin, which makes up the structural basis of a blood clot. Plasmin exists in its inactive form as plasminogen; the activation of plasminogen is mediated by serine enzymes known as tissue-type plasminogen activator (t-PA) and urokinase (u-PA). The proteolytic activity of t-PA and u-PA is, in turn, regulated by specific protease inhibitors, plasminogen activator inhibitor (PAI)-1 and PAI-2. Plasminogen deficiencies can also lead to thrombophilia in patients.

## Historical Background

A familial component of venous thrombosis was first fully recognized in the 1960s when reduced levels of AT were shown to be associated with recurrent thrombosis in a family [7]. Since this discovery, multiple studies have shown almost 250 different mutations for AT deficiency and the risks associated with this disorder. The next step in finding other causes of inherited thrombophilia followed 16 years later with the discovery of protein C deficiency [8]. The study by Broekman et al in 1983 of three Dutch families provided further understanding of this deficiency and confirmed the autosomal dominant inheritance pattern. That study of protein C demonstrated that inherited thrombophilia was a polygenic disorder with variable expressivity. This was followed a few years later with the discovery of an inherited deficiency of the co-factor for protein C, protein S. [9]. All three of these proteins, AT, protein C and protein S, play a role in the downregulation of coagulation. Deficiencies of these proteins result in an increased generation of thrombin and a predisposition to thrombosis.

Further confirmation of the multiple genetic factors for increased thrombotic risk came with the description of activated protein C-Resistance (APC-R) in 1993, and dramatically changed the diagnosis and management of venous thrombotic events. Dahlback et al described a large family from southern Sweden who demonstrated thrombosis in males and females throughout several generations and showed an autosomal dominant pattern of inheritance [10]. They observed a significant prolongation of the activated partial thromboplastin time (aPTT) in the normal plasma following the addition of APC (APC inactivates factor Va and factor VIIIa, thereby decreasing available thrombin). In contrast, plasma from the affected family showed a lack of significant prolongation of the APTT. The authors concluded that there was an abnormality in the protein C/protein S regulatory system. Subsequently, this abnormality was identified as a single amino acid substitution in one of the substrate proteins for APC: Factor V. This mutation was later characterized by Bertina and colleagues at the University of Leiden [11]. The rapidity with which the genetic mutation was identified following the phenotypic observation of APC-R is an example of the dramatic shift to gene-based diagnosis of disease. Dahlback et al subsequently reported that as many of 15% of the population in southern Sweden carried the factor V Leiden (FVL) gene. The identification of FVL significantly changed the way clinicians and laboratories approach the diagnosis of thrombophilia.

Early discoveries of genetic prothrombotic risk factors involved gene mutations that resulted in decreased concentrations or function of certain coagulation proteins. Eventually studies showed that elevations of proteins could also convey a risk for thrombophilia. For example, elevated Factor VIII has been described as a risk factor for recurrent venous thrombosis, especially when it is familial trait rather than an acute phase reactant [12]. Various other factors for congenital thrombophilia have been described including hyperhomocysteinemia, elevated lipoprotein and dysfibrinogenemia, to name a few. Studies have also shown that these factors may co-exist with other inherited defects leading to thrombophilia. As a consequence, there has been a marked change in the evaluation and management of thrombophilia in recent years. Table 1 provides a listing of established, rare and indeterminate factors causing genetic thrombophilia [13].

**Table 1:** Causes of genetic thrombophilia

**Established genetic factors**	**Rare genetic factors**	**Indeterminate factors**
Factor V Leiden	Dysfibrinogenemias	Elevated Factor VIII
Prothrombin G20210A	Hyperhomocysteinemia	Elevated Factor IX
Protein C deficiency		Elevated Factor XI
Protein S deficiency		Plasminogen deficiency
Antithrombin deficiency		Tissue plasminogen activator
		Elevated lipoprotein a
		Factor VII
		Factor XII
		Platelet glycoprotein
		Plasminogen activator inhibitor
		Heparin cofactor II
		Thrombomodulin
		Histidine-rich glycoprotein

## Epidemiology

Venous thrombosis has an overall annual incidence of < 1 in 1000. It is rare in the pediatric population, with rates of deep vein thrombosis of about 1 in 100,000 [1] and increases in frequency in older patients. While significant advances have been made in understanding congenital thrombophilias, there may still be many more heritable forms of thrombophilia as yet undiscovered. Thus, it is not possible to determine the true prevalence of congenital thrombophilia.

Pediatric disorders associated with genetic thrombophilia include neonatal purpura fulminans, renal vein thrombosis, vena cava thrombosis and hepatic venous thrombosis. Pulmonary embolism, Legg Calve Perthes and cerebral palsy have all been linked to genetic thrombophilia [13]. Complete deficiency of protein C or S (homozygous individuals) cause neonatal purpura fulminans and disseminated intravascular coagulation with an incidence of about 1 in 16,000–360,000 [14]. Table 2 shows the prevalence of genetic defects among Caucasians with venous thrombosis [15].

**Table 2:** Prevalence of genetic defects among Caucasians [15]

	Incident VTE (%)	Recurrent VTE (%)	Normal population	Relative thrombotic risk
Activated Protein C resistance (Factor V Leiden)	20	40–50	3–7	3–7 heterozygotes 50–100 homozygotes
Prothrombin G20210A mutation	3–8	15–20	1–3	2–8 heterozygotes
Antithrombin deficiency	1–2	2–5	0.02–0.04	5
Protein C deficiency	2–5	5–10	0.2–0.5	6–10
Protein S deficiency	1–3	5–10	0.1–1	2

A summary of the results from case series and case-control studies in caucasian children with venous thrombosis is presented in Table 3[16].

**Table 3:** The prevalence of risk factors among caucasian children with venous thrombosis: a summary of the results from case series and case-control studies[16].

	**Controls**	**Patients**	**Odds ratio (95% CI)**
Factor V Leiden	15/370	83/261	11 (6.2–19.7)
Prothrombin G20210A	4/370	11/261	4.1(1.3–12.8)
Protein C deficiency	3/370	24/261	12.4 (3.7–41.6)
Protein S deficiency	3/370	15/261	7.5 (2.1–26)
Antithrombin deficiency	0/370	9/261	--
Lipoprotein a > 30 mg/dl	19/370	78/261	7.2

Thromboembolic events cause significant mortality and morbidity among patients of all ages. Possibly owing to the lower concentrations of antithrombin, heparin cofactor II, and protein C, along with a reduced fibrinolytic capacity, neonates are at greater risk of thromboembolic complications than older children [16]. The incidence of vascular accidents decreases significantly after the first year of life, with a second peak during puberty and adolescence, again associated with reduced fibrinolytic activity. The increased understanding of thrombophilias at a molecular level that has developed over the past 40 years has lead to conceptual changes on how to diagnose and manage the disorder. Studies have been done in various populations to understand the inheritance patterns and risks for individuals with an inherited thrombophilia. Familial thrombosis was originally considered an autosomal dominant disorder with varying expression and penetrance. However, more recent studies suggest that congenital thrombophilia may in fact be the result of the combination of two or more gene defects in a family [17]. The following sections will discuss the various congenital thrombophilias and the degree to which each of these disorders puts an individual at risk for a thromboembolic event.

## Specific types of inherited thrombophilias

### 1. Factor V Leiden

In 1994, Bertina et al first described a defect in the factor V gene that makes it less susceptible to inactivation by activated protein C (APC). The following year, Kalafatis et al showed the mechanism of inactivation of the membrane bound profactor Va is an ordered event. Factor Va is sequentially cleaved at Arg506 and at Arg306 and Arg679 by activated protein C [18]. They suggested that the peptide bond cleavage at Arg506 facilitates the exposure of the subsequent cleavage sites at Arg306 and Arg679. At around the same time, Shen and Dahlback et al showed that factor V is also a cofactor in the inactivation of factor VIIIa by APC [19].

The understanding of factor V inactivation was almost immediately followed by reports on how activated protein C in patients' plasma failed to prolong the activated partial thromboplastin time, hence the term "activated protein C resistance" was developed [20].

Further studies have shown that most patients with activated protein C resistance have a factor V allele that is resistant to the proteolytic effect of protein C. A transition (guanine to adenine) at nucleotide 1691 (G1691) results in the replacement of arginine by glutamine. This gene product, called factor V Leiden, also known as factor V Q506 or Arg506Gln, is named after the city in the Netherlands that it was first identified in. Factor V Leiden is a variant of the normal gene and is not susceptible to cleavage at position 506 by activated protein C. The consequence of this is a hypercoagulable state as more factor Va is available within the prothrombinase complex, thereby increasing the generation of thrombin. Factor V is also thought to be a cofactor, along with protein S, in supporting the role of activated protein C in the degradation of factors Va and VIIIa. Thus, lack of this cleavage product decreases the anticoagulant activity of activated protein C.

Several mutations at the Arg306 residue in factor V have been described in patients with a history of thrombosis. These include replacement of Arg306 with threonine (factor V Cambridge) [21] or with glycine (in Hong Kong Chinese)[22]. Occasionally, patients have been described who have heterozygous APC resistance due to the factor V Leiden mutation and type I factor V deficiency [23]. The plasma of these individuals manifests severe APC resistance in activated partial thromboplastin time assays, similar to that seen in patients with homozygous factor V Leiden. These patients appear to be more thrombosis prone than their heterozygous relatives with factor V Leiden alone, suggesting that the clinical phenotype is similar to patients who are homozygous for factor V Leiden.

#### Factor V Leiden and risk of thrombosis

There are multiple studies showing evidence for factor V Leiden as a cause of deep vein thrombosis (DVT) among the Caucasian population. The major clinical manifestation is deep vein thrombosis with or without pulmonary embolism. There is also evidence that the factor V Leiden mutation, presumably due to thrombosis of placental vessels, may play a role in some cases of unexplained recurrent pregnancy loss.[24]

Svennson and Dahlback studied 34 families with the Factor V506 Arg to Gln mutation and found an increased lifetime risk of venous thrombosis. By age 50, at least 25% of those affected had experienced at least one thrombotic event. The Leiden Thrombophilia Study by Koster et al in the Netherlands provided a population-based case control study to assess the prevalence of this disorder. APC-R was found in 21% of those who had a history of thromboembolism compared with 5% of controls. Overall, the relative risk for a thromboembolic event was increased seven-fold with heterozygous individuals. They further studied individuals who were homozygous for factor V Leiden mutation and found an 80-fold increase in the lifetime risk for a thrombotic problem. It was subsequently estimated that homozygous individuals can be expected to experience at least one venous thromboembolic event in their lifetime [20]. This is supported by a study of 306 family members from 50 Swedish families, which found 40 percent of homozygotes had an episode of venous thrombosis by age 33, compared to 20 percent of heterozygotes and 8 percent of normals.

Ridker et al published a study in 1997 based on 4047 American men and women participating in the Physician's Health Study and the Women's Health Study. The study found a 12 percent incidence of heterozygosity for the factor V Leiden mutation in patients with a first confirmed deep vein DVT or pulmonary embolism compared with 6 percent in controls [24]. The incidence reached 26 percent in men over the age of 60 with no identifiable precipitating factors.

#### Factor V Leiden in different populations

The prevalence of heterozygosity for the factor V Leiden mutation in Europeans, Israeli, Arab, Canadian and Indian populations, ranges from 1 to 8.5 percent with most European studies reporting overall rates between 5 and 8 percent. The prevalence is highest in Greece, Sweden, and Lebanon where it approximates 15 percent in some areas. On the other hand, the mutation is apparently not present in African Blacks, Chinese, or Japanese populations [25].

Although possibly influenced by a selection bias, the lifetime probability of developing thrombosis is considerably less in heterozygotes with the factor V Leiden mutation than in patients with the less common inherited thrombophilias. This was illustrated in a report which compared the risk for thrombosis in a 150 Italian individuals with inherited thrombophilia due to factor V Leiden in relation to those with antithrombin, protein C, or protein S deficiency [26]. The lifetime probability of developing thrombophilia was 8.5 times higher for carriers of protein S deficiency, 8.1 for antithrombin deficiency, 7.3 for protein C deficiency, and 2.2 for factor V Leiden when compared to controls.

An extensive review of different populations was conducted by Rees et al in 1995 where they collected genetic data from 1690 unrelated individuals from 24 different populations [27]. Unfortunately, the study does not clarify the methods for the selection of the individuals nor the inclusion/exclusion criteria. The study does provide a breakdown for both heterozygotes and homozygotes of the factor V Leiden mutation and shows significant differences among populations. The study confirms a higher prevalence of the mutation among Europeans compared to individuals from other parts of the world.

### 2. Prothrombin gene mutation

In 1996, Poort et al described single amino acid genetic variation in the 3' untranslated region of the gene that codes for prothrombin. Prothrombin (factor II) is the precursor to thrombin, the end-product of the coagulation cascade. Prothrombin has procoagulant, anticoagulant and antifibrinolytic activities and thus a disorder involving prothrombin results in multiple imbalances in hemostasis. A report published in 1996 based on 28 families from the Netherlands with established venous thromboembolism identified a substitution of guanine to adenine at nucleotide 20210 in the 3' untranslated region of the prothrombin gene [28]. Linkage studies performed in 397 individuals from 21 Spanish families have provided further evidence that a quantitative trait locus (G20210) in the prothrombin gene influences prothrombin activity levels and susceptibility to thrombosis [29].

#### Prothrombin gene mutation and risk of thrombosis

The prothrombin G20210A gene mutation is associated with an elevated risk of deep vein thrombosis, although to a lesser degree than factor V Leiden is. The Leiden Thrombophilia Study, a population-based study demonstrated a prevalence of the G20210A allele among healthy carriers to be 6.2% among venous thrombosis patients and 2.3% among healthy matched controls. The study also showed that among heterozygotes with the prothrombin gene mutation, 87% of thrombosis patients in the study had prothrombin activity levels that were > 1.15 U/ml, whereas only 23% of healthy individuals had levels that were elevated to this degree [20].

In 1996 Poort et al compared individuals from 28 families from the Netherlands with venous thrombosis with 100 normal adults and showed an odds ratio of 2.8 (95% CI 1.4–5.6) for those with the G20210A allele. Their results demonstrated that elevated prothrombin and the presence of allele G20210A were both risk factors for thrombosis. Heterozygous carriers were shown to have 30 percent higher plasma prothrombin levels than normal individuals. There is wide variability in geographic distribution of the prothrombin gene mutation. The proportion of white individuals heterozygous for the allele varies from 0.7 percent to 6.5 percent, with the highest prevalence rates reported in Spain [30].

A review of data from 11 centers in Europe found a range of 0.7 to 4.0 percent, with the prevalence in southern Europe being almost twice as high as northern Europe (3.0 versus 1.7 percent). The prothrombin gene mutation so far has been shown to be extremely rare in the nonwhite (black or Asian) population. Zilvelin et al postulated a "founder effect", suggesting the mutation probably occurred after the divergence of Africans from non-Africans and of Caucasians from Mongoloid subpopulations [31].

### 3. Protein C deficiency

Protein C deficiency is less common than either the factor V Leiden or the prothrombin G20210A gene mutation with prevalence in Caucasians estimated to be 0.2–0.5% [2]. Protein C deficiency is inherited in an autosomal dominant manner and is associated with familial venous thrombosis. The gene for protein C is located on chromosome 2 (2q13–14) and appears to be closely related to the gene for factor IX [32]. The primary effect of activated protein C (APC) is to inactivate coagulation factors Va and VIIIa, which are necessary for efficient thrombin generation and factor X activation [33]. The inhibitory effect of APC is markedly enhanced by protein S, another vitamin K-dependent protein.

Two major subtypes of heterozygous protein C deficiency (Type I and Type II) have been delineated using immunologic and functional assays. Over 160 different gene abnormalities have been associated with the two subtypes [34].

Type I deficiency – The type I deficiency state is more common. Most affected patients are heterozygous, having a reduced plasma protein C concentration at approximately 50 percent of normal in both immunologic and functional assays [35]. More than half of the mutations identified so far are missense and nonsense mutations. Other types of mutations include promoter mutations, splice site abnormalities. in-frame deletions, frameshift deletions, in-frame insertions, and frameshift insertions [34]. There is marked phenotypic variability among patients with heterozygous type I protein C deficiency. Similar mutations have been found among symptomatic and asymptomatic individuals. This finding suggests that the nature of the protein C gene defect alone does not explain the phenotypic variability.

Type II deficiency – Individuals with the type II deficiency state have normal plasma protein C antigen levels with decreased functional activity. A variety of different point mutations affecting protein function have been identified in this disorder [34].

#### Protein C and risk of thrombosis

Although the clinical manifestations of protein C are similar to those of antithrombin deficiency, there are some unique features of protein C deficiency. In a study of 11 infants in Denver, Colorado in 1988, Manco-Johnson et al suggested that homozygotes can develop a severe thrombotic tendency in infancy characterized as purpura fulminans [36]. Heterozygotes for protein C deficiency have an increased risk of developing warfarin-induced skin necrosis [37]. Protein C deficiency has been implicated in adverse pregnancy outcomes such as DVT, preeclampsia, intrauterine growth restriction and recurrent pregnancy loss [38].

Family studies from the Netherlands and the US have shown that family members who are PC deficient are at an 8–10 fold increased risk of venous thrombosis, and, by age 40, 50% or more will have experienced a thrombotic event [39,35]. The initial episode of venous thromboembolism in patients with protein C deficiency is apparently spontaneous in approximately 70 percent of cases. The remainder of the cases suggest that other genetic or acquired factors are involved in the presentation of thrombotic events in this population. Further studies in the Netherlands showed that most patients are asymptomatic until their early twenties, with increasing numbers experiencing thrombotic events as they reach the age of 50 [40]. Lensen et al concluded that the median age at onset for a thrombotic event and the risk of thrombosis is similar in both protein C deficiency and factor V Leiden (APC resistance). Approximately 60 percent of affected individuals develop recurrent venous thrombosis and about 40 percent have signs of pulmonary embolism.

The first case-control study looking at protein C deficiency was conducted by Heijboer et al in 1990 who did a study on 277 Dutch patients and 138 controls. The overall prevalence of protein C deficiency in the patients with venous thrombosis was 8.3 percent (23 of 277 patients) (95% CI 5.4–12.4), as compared with 2.2 percent in the controls (i.e. 3 of 138 subjects 95% CI 0.5–6.1; P < 0.05). Interestingly, the relative risk (RR) estimate for thrombosis given protein C deficiency was also close to 7 as in the case of the family studies in Netherlands.

Both the work done by Heijboer et al and a subsequent study by Tait et al estimated the population prevalence of heterozygous protein C deficiency at 0.2% [41]. The relative risk for thrombosis among patients with protein C deficiency was carried out by Koster et al involving 474 consecutive outpatients at anticoagulation clinics who were excluded by age (greater than 70) and known malignancy. The patients were asked to find their own controls by sex, age and no known thrombotic disorder. The study demonstrated the relative risk of thrombosis with protein C deficiency to be at least 3.1 (95% CI 1.4–7) with reevaluation of patients with low levels increasing the RR to closer to 4 [42].

There is marked variability in risk among families with protein C deficiency that cannot be explained by the genetic defect itself. In severely affected families, as many as 75 percent of protein C-deficient individuals experience one or more thrombotic events [43]; in other families, the thrombosis rate is much lower. A risk factor for more severe disease is the presence of a second thrombotic defect, particularly factor V Leiden as will be discussed in greater detail later on in the paper.

### 4. Protein S deficiency

Protein S serves as a cofactor for activated protein C. It was originally discovered and purified in Seattle, leading to the designation protein S. There are two homologous genes for protein S: PROS1 and PROS2, which both map to chromosome 3 [44].

In 2000, Gandrille et al identified 15 point mutations and 3 polymorphisms among 19 French patients; since then mutations have been identified in 70 percent of protein S deficient probands and a database of known protein S gene mutations has been published [45]. As in the case with the other inherited thrombophilias, there seem to be few reports of large deletions causing the disorder. Three phenotypes of protein S deficiency have been defined on the basis of total protein S antigen concentrations, free protein S concentrations, and protein S functional activity.

Type I – The classic type of protein S deficiency is associated with a decreased level of total S antigen (approximately 50% of normal), and marked reductions in free protein S antigen and protein S functional activity [46].

Type II – This type of protein S deficiency is characterized by normal total and free protein S levels, but diminished protein S functional activity. Interestingly, all five mutations originally described in these patients were missense mutations located in the N-terminal end of the protein S molecule, which includes the domains that interact with activated protein C [47].

Type III – Also known as type IIa, this is characterized by total protein S antigen measurements in the normal range and selectively reduced levels of free protein S and protein S functional activity to less than approximately 40 percent of normal [48]. Interestingly, a case-control study by Zoller in 1995 involving 327 Swedish families showed that type I and type III are phenotypic variants of the same genetic disease.

#### Protein S deficiency and risk of thrombosis

Protein S deficiency is inherited in an autosomal dominant manner and is at least as common as antithrombin and protein C deficiency [5]. The clinical manifestations are similar to those seen with antithrombin and protein C deficiency. Thrombosis occurs in heterozygotes whose levels of functional protein S are in the range of 15–50% of normal.

The prevalence of familial deficiency of protein S type I among Caucasians, estimated from a large cohort of healthy blood donors from the West of Scotland, is in the range of 0.03 to 0.13 percent [49]. The prevalence is much higher among individuals with established thrombophilia. In a Spanish study of 2132 consecutive unselected patients with venous thromboembolism, 7.3 percent had protein S deficiency [50]. Based on these and other studies, Martinelli et al estimated the lifetime probability of developing thrombosis among carriers of protein S deficiency was 8.5 times higher compared to those with no defect.

In 1987, Engesser and colleagues conducted a study on 12 Swedish families with 136 members and found 71 of them to be heterozygous for Type I protein S deficiency. 55% of those who carried the defect were found to have had a thrombotic event and 77% of those were recurrent. About half of the cases were precipitated by another condition. They also showed that in phenotypic protein S deficient families, the likelihood that affected family members remain thrombosis-free at 45 years of age was 35 to 50 percent. This study showed a difference in rates between men and women but was not able to provide an adequate explanation in terms of difference in risk factors between the two sexes [51].

Another study examined the incidence of thrombosis in carriers in a Swedish family with a known missense mutation (Gly295Val) [52]. Simmonds studied 122 members in a family, with 44 of the members previously characterized for the specific gene defect in protein S. The study showed little thrombotic risk before the age of 15 years. On the other hand, the likelihood of being free of thrombosis by age 30 was only 50 percent compared to 97 percent in normal family members. The odds ratio for thrombosis in affected subjects was 11.5, and the study showed that measurement of free protein S antigen levels was predictive of the mutation and deficiency. In a UK based family study with 28 index patients with protein S deficiency, first degree relatives with the PROS1 gene defect had a five-fold higher risk of thrombosis than those with a normal gene and no other apparent thrombophilia [53].

Overall, both family and cohort studies demonstrate that like other thrombophilic disorders, heterozygous protein S deficiency usually manifests in adulthood with a thromboembolic event. When present with other thrombophilias or when present in the homozygous form, protein S usually presents in neonates with purpura fulminans [54].

### 5. Antithrombin deficiency

Antithrombin (AT) deficiency is a heterogeneous disorder. AT It is usually inherited in an autosomal dominant fashion, thereby affecting both sexes equally. The first database for antithrombin gene mutations was published in 1991 and this has subsequently undergone various revisions as newer mutations have been described. There are different AT deficiencies based on the subtypes, as follows:

Type I – The type I deficiency state results from reduced synthesis of biologically normal protease inhibitor molecules [55]. In these cases both the antigenic and functional activity of AT in the blood are reduced. The values are reduced by approximately 50 percent in the heterozygote. The 1997 antithrombin mutation database included 80 distinct mutations in patients with type I deficiency [56]. The database shows that the molecular basis for this disorder is usually a small deletion or insertion (less than 22 base pairs) or a deletion of a major segment of the AT gene.

Type II – Type II AT deficiency is produced by a discrete molecular defect within the protein. While the AT immunologic activity is normal in this deficiency, plasma AT functional activity is markedly reduced leading to risk of thrombosis.

Type III – This type is characterized by normal functional and antigenic antithrombin levels but impaired interaction between AT and heparin [41].

*Heparin binding site defect *– Several abnormal AT molecules have been identified with defects at a heparin binding site, resulting in isolated reductions in heparin cofactor activity. These variants generally have mutations at the amino-terminal end of the molecule [3]. Affected individuals generally have plasma AT-heparin cofactor activity measurements of approximately 50 percent and normal progressive AT activity.

#### AT deficiency and risk of thrombosis

Congenital antithrombin deficiency is inherited as an autosomal dominant trait. Affected patients have antithrombin levels 40–60% of normal, and 70% of those affected experience thrombo-embolic events before the age of 50. Thrombotic episodes are rare before puberty in AT-deficient individuals. They start to occur with some frequency after puberty, with the risk increasing substantially with advancing age [57].

In 1994, Tait et al studied 9,669 blood donors in Scotland by taking blood samples and monitoring the donors for two years. Of the study participants, 107 were found to have an initial level of AT < 83 iu/dl. They were retested, and eventually, 16 donors where found to have congenital AT deficiency. The study suggested 1/600 or 1.65 per 1000 (95% CI of 0.95–2.27 per 1000). 2 of the affected individuals had Type I deficiency while the other 14 had Type II. Making corrections for donors who defaulted, the study showed an overall prevalence of 1/4400 for Type I AT deficiency and 1/630 for type II with an overall prevalence of AT deficiency at 1/630 in the population studied. The individuals with AT > 83 iu/dl after being retested is explained by normal intra-individual and inter-assay variation, but nevertheless brings into question the accuracy of the testing. The authors themselves considered another round of testing, but realized that that was not practical with the large number of people tested. While selection bias is obviously present, the authors felt that this was minimal as their results were comparable to smaller studies done previously. They bring up the important issue of whether the AT deficiency itself, or in combination with other factors, would make a thromboembolic event likely in these individuals. Another challenge when testing for AT without other clinical information is that levels of antithrombin can be reduced in protein-losing states. Marked reductions in antithrombin levels occur in patients with liver disease as well as severe malnutrition and these can cause skewed results [58].

Using only an immunoassay for measuring plasma levels of AT, initial estimates of the prevalence of AT deficiency in the general Scottish population were one in 2000 to 5000. However, studies employing functional assays that measure AT-heparin cofactor activity have found that the prevalence of AT deficiency in the Scottish population is one in 250 to 500 [41]. The majority of AT-deficient patients identified in these studies did not have a personal or familial history of thrombosis and had a type II defect with mutations at the heparin binding site. Among patients with a first thrombotic event, the prevalence of hereditary AT deficiency is approximately 0.5 to 1 percent, being less common than factor V Leiden, the prothrombin gene mutation, or protein S/protein C deficiency. A recent study showed that homozygous children of consanguineous parents who were asymptomatic carriers developed severe venous or arterial thrombosis in association with plasma AT-heparin cofactor levels below 10 percent of normal [59].

The thrombotic risk associated with AT deficiency, as with other inherited thrombophilias, has been assessed in two ways: evaluation of patients with deep vein thrombosis and evaluation of families with thrombophilia. In a Spanish study of 2132 consecutive unselected patients with venous thromboembolism 12.9 percent had an anticoagulant protein deficiency (7.3 percent with protein S, 3.2 percent with protein C, and 0.5 percent with antithrombin). Similar findings were noted in a series of 277 Dutch patients with deep vein thrombosis: 8.3 percent had an isolated deficiency of antithrombin, protein C, protein S, or plasminogen compared to 2.2 percent of controls [5]. In a study in 2001, five children from three Austrian families had a homozygous antithrombin deficiency type II affecting the heparin binding site (99 Leu to Phe mutation). Four children had severe spontaneous thromboembolic events (deep leg or caval vein thrombosis, ischemic stroke) at one week, 3 months, 13 and 14 years of age. The fifth patient, a 17 year-old boy was asymptomatic [60].

The absolute risk of thrombosis among patients with inherited thrombophilia was evaluated by Martinelli et al in 1998 in an Italian cohort study of 150 pedigrees consisting of 1213 individuals. The study compared the risk for thrombosis in individuals with inherited thrombophilia due to factor V Leiden, antithrombin, protein C, or protein S deficiency [26]. The lifetime probability of developing thrombosis compared to those with no defect was 8.5 times higher for carriers of protein S deficiency, 8.1 for type I antithrombin deficiency, 7.3 for protein C deficiency, and 2.2 for factor V Leiden. The selection of patients was not solely based on their registration at the thrombosis centers in Milan or Rome but also required individuals to provide clinical evidence of thrombotic events and hence increases the validity of the study.

### 6. Dysfibrinogenemia

The abnormal production of fibrinogen can result in dysfibrinogenemia. The abnormal fibrinogen usually exhibits an abnormal thrombin-mediated conversion to fibrin. In 1984, Lijnen et al reported that the fibrin-mediated enhancement of the activation of plasminogen by tissue-type plasminogen activator observed with normal fibrin is strongly decreased with the abnormal type known as fibrin Dusard, although the binding of tissue-type plasminogen activator to the fibrin was normal. They suggested that this impaired fibrin-mediated plasminogen activation is most likely the cause of thrombophilia among affected patients [61]. Over 300 examples from unrelated families have been reported in the literature. Over 90 percent of these cases are point missense mutations, leading to the production of a dysfunctional protein product. A website by Hanss and Biot started in 2001 maintains an updated version of all fibrinogen variants with the last update showing 330 different mutations [62]. Congenital dysfibrinogenemias are named after the city where the patient was first identified or evaluated. Roman numerals are added after the city name when there are several dysfibrinogenemias from the same city (e.g., Caracas V). With rare exceptions, the mode of inheritance of the congenital dysfibrinogenemias is autosomal dominant.

#### Dysfibrinogenemia and the risk of thrombosis

While most patients with dysfibrinogenemia are clinically asymptomatic, some present with a bleeding diathesis, others with thrombophilia, and occasionally with both, bleeding and thromboembolism. A study in 1990 by Heijboer et al in this last group (i.e. those with both bleeding and thromboembolism) showed the risk of thrombosis in affected family members is much higher than in those who are unaffected, suggesting that there is a true relationship between certain dysfibrinogenemias and thrombosis. A meta-analysis study looking at 9 studies put together the various reported cases from the USA and different countries in Europe. They were unable to show a clear connection between dysfibrinogenemia and thrombosis [63]. A study in Italy in 2000 demonstrated that missense mutations in the beta-fibrinogen gene could cause congenital afibrinogenemia by impairing fibrinogen secretion [64]. However, the exact mechanism of how abnormal fibrin results in thrombosis is yet to be elucidated.

The prevalence of congenital dysfibrinogenemia in patients with a history of venous thrombosis has been estimated at 0.8 percent. The true prevalence of thrombosis among patients with dysfibrinogenemia is unknown, but is estimated to be around 10 to 20 percent [63].

### 7. Hyperhomocysteinemia

Hyperhomocysteinemia may be both a genetic and acquired abnormality. Homocystinuria and hyperhomocysteinemia can be caused by rare inborn errors of metabolism that result in marked elevations of plasma and urine homocysteine concentrations.

Homocysteine is an amino acid derived from methionine. Hyperhomocysteinemia occurs when increased amounts of the amino acid accumulate in the blood due to of impaired intracellular metabolism of homocysteine. Homocysteine is metabolized by the body in two possible pathways: transsulfuration, and remethylation. The transsulfuration of homocysteine produces cysteine and the reaction is catalyzed by cystathionine-β-synthase. This process requires pyridoxal phosphate (Vitamin B) as a cofactor. Remethylation of homocysteine produces methionine. This reaction is catalyzed either by methionine synthase or by betaine-homocysteine methyltransferase. Vitamin B12 (cobalamin) is the precursor of methylcobalamin, which is the cofactor for methionine synthase. Elevations in the plasma homocysteine concentration can occur due to genetic defects in the enzymes involved in homocysteine metabolism as well as due to nutritional deficiencies in vitamin cofactors, or to other factors including some chronic medical conditions and drugs.

The most common form of genetic hyperhomocysteinemia results from production of a thermolabile variant of methylene tetrahydrofolate reductase (MTHFR) with reduced enzymatic activity (T mutation) [65]. The gene encoding for this variant contains an alanine-to-valine substitution at amino acid 677 (C677T) [66].

## Homocysteinemia and the risk of thrombosis

In 1969, McCully demonstrated that premature atherosclerosis and arterial thrombosis is associated with severe hyperhomocysteinemia [67]. Subsequent investigations have confirmed this hypothesis, and it has recently become clear that hyperhomocysteinemia is an independent risk factor for thrombosis. Although severe hyperhomocysteinemia is rare, mild hyperhomocysteinemia occurs in approximately 5 to 7 percent of the general population [68].

In 1997 D'Angelo et al suggested that the genetic defect may be present in 1.4–15% of Caucasians [69]. A meta-analysis of 40 observational studies involving 11,162 patients who were homozygous for the thermolabile variant of MTHFR and 12,758 matched controls demonstrated that patients with the MTHFR TT genotype had a 16 percent higher odds of coronary heart disease compared with controls (odds ratio 1.16, 95% CI 1.05–1.28).

Den Heijer and colleagues have demonstrated that mild hyperhomocysteinemia is an independent risk factor for venous thromboembolism [70]. They found a marked increase in the risk of venous thrombosis at the highest plasma homocysteine concentrations. In their case-control study of 269 patients with a first, objectively diagnosed episode of deep vein thrombosis and 269 healthy controls, 28 had plasma homocysteine levels above the 95th percentile for the controls, as compared with 13 of the controls with a matched odds ratio of 2.5; 95 % CI: 1.2–5.2. They showed that a plasma homocysteine concentration of more than 22 μmol per liter increased the matched odds ratio for deep venous thrombosis to 4.0. Recently, Ridker et al showed that the combination of hyperhomocysteinemia and factor V Leiden further increases the relative risk of venous thromboembolism up to 3.6-fold [71].

To investigate if homocysteine is a modifiable risk factor, Bonna studied the effect of supplementation on homocysteine levels as part of the Heart Outcomes Prevention Evaluation (HOPE 2) investigation [72]. The study showed that supplements combining folic acid and vitamins B_6 _and B_12 _did not reduce the risk of major cardiovascular events in patients with vascular disease. Bonna also suggested that homocysteine could be a marker, but not a cause, of vascular disease. At the same time, Loscalzo pointed out that while folic acid, vitamin B_12_, and vitamin B_6 _change the homocysteine levels there is likely a complex metabolic interactions involved that will need to be explored further before any conclusions can be drawn regarding homocysteine and the risk of thrombosis [73]

While there is evidence that hyperhomocysteinemia is also a risk factor for venous thromboembolism (VTE, there are conflicting data as to whether the risk of VTE is markedly increased in patients when hyperhomocysteinemia is combined with an inherited thrombophilia [74].

### 8. Factor VII

In the coagulation pathway the association of factor VIIa with tissue factor enhances the proteolytic activity by (1) bringing the binding sites for both the substrate (factors X and IX) and the enzyme (VIIa) into closer proximity and by (2) inducing a conformational change, enhancing the enzymatic activity of factor VIIa. There are several studies that demonstrate that deficiency of factor VII can cause significant increase in bleeding risk among affected individuals. There is also some evidence that elevated Factor VII is related to an increased risk of thrombosis. The Northwick Park Heart Study was a prospective study in the early 1990s in which factor VII levels were found to be strongly associated with thrombotic risk. The study showed that fibrinolytic activity as measured by dilute blood clot lysis time was associated with elevated factor VII and increased the risk of fatal events among affected patients [75].

### 9. Elevated Factor VIII

Elevated plasma factor VIII coagulant activity (VIII:C) is now accepted as an independent marker of increased thrombotic risk. In a population-based, case-control study performed in the Netherlands by Koster et al in 1995, individuals with factor VIII:C levels greater than 150 percent of normal had an adjusted odds ratio of 4.8 for a first episode of a venous thrombosis event compared to individuals with levels under 100 percent. In both this study and an English study involving 46 patients referred for evaluation of unexplained thrombosis, the incidence of elevated factor VIII:C levels was approximately 25 percent [76]. In a case-control study of 185 Dutch individuals, Kraaijenhagen et al looked at different levels of factor VIIIc and the associated risk of thrombosis. They found an odds ratio of thromboembolism for factor VIIIc > 200IU/dl as high as 11 (95%CI 2–71) for a single episode and 45 (95% CI: 6–370) for recurrent episodes of thromboembolism. The study demonstrated factor VIIIc as an independent risk factor for thrombosis. Kraaijenhagen et al also suggested there was a direct relationship between the degree of increase of factor VIII and the risk of thrombosis [77].

### 10. Elevated Factor IX

The previous section described how elevated plasma levels of factor VIII (> 150 IU/dL) are an important risk factor for deep vein thrombosis. Factor VIII is the cofactor of factor IXa in the activation of factor X and hence, it has been postulated that elevated levels of factor IX can also increase the risk of thrombosis [78]. Factor IX is a circulating serine protease that serves as an essential component of the blood coagulation pathway, and has been shown to increase with age in humans [79]. A study was conducted in 2000 involving 426 Dutch patients with a first, objectively-diagnosed episode of DVT compared to 473 population controls. This study was part of a large population-based case-control study on risk factors for venous thrombosis, the Leiden Thrombophilia Study. Using the 90th percentile measured in control subjects (P[90] = 129 U/dL) as a cutoff point for factor IX levels, van Hylckama Vlieg et al found a 2- to 3-fold increased risk for individuals who have factor IX levels above 129 U/dL compared with individuals having factor IX levels below this cutoff point [80]. After exclusion of individuals with known genetic disorders, they found an odds ratio of 2.5 (95% confidence interval: 1.6–3.9). These results show that an elevated level of factor IX is a common risk factor for thrombosis among the Dutch population.

### 11. Elevated Factor XI

Factor XI, a component of the intrinsic pathway of coagulation, contributes to the generation of thrombin, which is involved in both the formation of fibrin and protection against fibrinolysis. Looking at genetic data from the Leiden Thrombophilia Study based in the Netherlands, using 474 cases and 474 controls, the authors compared individuals who had a factor XI level above the 90^th ^percentile to those who had levels below that level. The age- and sex-adjusted odds ratio for deep venous thrombosis was found to be 2.2 (95 % CI: 1.5–3.2). As is the case with factor VIII, this study suggested that the degree of elevation of factor XI correlates with risk of venous thrombosis [81].

### 12. Factor XII deficiency

Factor XII or Hageman factor (named after the first patient found to have this deficiency) is the zymogen of a serine protease that initiates the contact activation reactions and intrinsic blood coagulation in vitro. Severe factor XII deficiency (factor XII activity less than 1 percent of normal) is inherited as an autosomal recessive trait. Affected patients have marked prolongation in the activated partial thromboplastin time (aPTT) and increased thrombotic tendency likely due to reduced plasma fibrinolytic activity. Evaluating 14 Swiss families in 1991, Lammle et al demonstrated that homozygous factor XII deficiency may be associated with an increased risk for venous thromboembolic disease. However, they suggested that partial factor XII deficiency was not, by itself, a strong risk factor for thrombosis [82]. In 2004, Girolami et al looked at reported cases of homozygous factor XII deficiency among Italian patients and noted 11 cases of venous thrombosis had been described. All but four of the cases were noted to be associated with various other risk factors such as pregnancy, the postpartum period, surgery, trauma, AT deficiency, or, heterozygous factor V Leiden. They concluded that the role played by FXII deficiency in the pathogenesis of venous thrombosis is minor, if any [83].

### 13. Elevated Lipoprotein a

Lipoprotein a (Lp a) is an inherited risk factor for thromboembolism [84]. Lp (a) inhibits the binding of plasminogen to the cell surface, reducing plasmin generation and subsequent clot lysis. In 2001, Caplice et al demonstrated that along with its antifibrinolytic mechanism of action, Lp (a) also binds and inactivates TF-pathway inhibitor, which is a major endogenous regulator of tissue factor (TF)-mediated coagulation [85]. A case controlled study based on Germany by von Depka et al in 2000 of 603 patients and 430 healthy adults showed an independent association between elevated levels of lipoprotein a (> 300 mg/L) and venous thromboembolism (95% CI: 1.4–3.2 with p value 0.002) [86]. Another study in Germany around the same time showed that 186 children with a history of venous thrombosis had a significantly higher median Lp(a) level (19 versus 4.4 mg/dL) than the same number of control subjects. The risk for thromboembolic events in children with Lp(a) levels in the upper quartile, ie, > 30 mg/dL, was 7.2 (95% CI, 3.7 to 14.5) [87].

### 14. Platelet glycoprotein gene polymorphisms

Being a critical element of the clot forming process, platelets and platelet glycoprotein gene polymorphisms have received increasing attention as possible inherited determinants of prothrombotic tendency. However, their role in genetic susceptibility to thrombotic disease remains controversial. Reiner et al suggested in 2001 that the glycoprotein IIIa Leu33Pro amino acid substitution was associated with a subtle effect on platelet thrombogenicity in vitro. The Leu33Pro allele seems to cause structural changes in the GPIIb/IIIa receptor which may in turn lead to a different ligand binding of the active GPIIb/IIIa receptor. However, Reiner et al concluded that it is not a major risk factor for arterial thrombotic disease among the general population [88]. Platelet collagen receptor (glycoprotein Ia/IIa; integrin alpha2 beta1) polymorphisms have also been implicated in thrombotic disease [89]. More studies need to be done to establish a relationship between the risk of thrombotic events and platelet glycoprotein gene polymorphisms.

### 15. Plasminogen Deficiency

Two major components of the fibrinolytic system are plasminogen and tissue-type plasminogen activator (tPA). Plasminogen is converted to the active enzyme plasmin by tPA in the presence of fibrin. Plasmin, in turn, digests the fibrin clot, forming soluble fibrin degradation products. A deficiency of either tPA or plasminogen could reduce the capacity to remove excessive clot and contribute to thromboembolic disease [90].

In 1978, Aoki et al identified the first case of an abnormal plasminogen [91]. In a large multi-center study in 1997, Mateo et al looked at plasminogen levels in over 2000 Spanish patients presenting with venous thrombosis. Their studies suggested that plasminogen deficiency occurs in 0.5% to 2.0% of patients with a history of thrombosis, a frequency similar to that of antithrombin deficiency in the same populations. On the other hand, in a survey of 53 Swiss family members of 23 plasminogen-deficient patients, Demarmels et al found that 5 (18%) of 28 family members with plasminogen deficiency had a history of thrombosis, whereas 5 (20%) of 25 family members with normal plasminogen levels also had a history of thrombosis. Although there is no apparent difference in the rate of thrombosis between those with and without plasminogen deficiency, the overall rate of thrombosis in family members in this study was high. This could be in part due to the presence of additional risk factors for thrombosis, such as factor V Leiden [92].

There are both quantitative defects (type I, hypoplasminogenemia in the heterozygote and aplasminogenemia in the homozygote) and functional defects (type 2, dysplasminogenemia) with plasminogen deficiency. Thrombosis has been reported in young patients when the plasminogen concentration is less than 40 percent of control values. The development of ligneous conjunctivitis, hyperviscosity of tracheobronchial and nasopharyngeal secretions, impaired wound healing, and hydrocephalus soon after birth have been reported in a German patient with homozygous plasminogen deficiency [93].

### 16. Tissue Plasminogen Activator (tPA)

In a multi-center, randomized trial in 1996, Schulman and Wiman evaluated the relationship between various fibrinolytic parameters and the incidence of recurrent thrombosis among nearly a thousand Swedish patients with venous thrombosis [94]. They noted that there was no significant difference in the absolute values of tPA between those patients who did and did not have recurrent thrombosis. However, a higher percentage (50% vs 36%, *P *< .001) of patients in the group with recurrent thrombosis had a tPA level above the upper limit of normal (10 ng/mL) than in the patients without recurrent thrombosis. The authors concluded that increased levels tPA antigen in patients with venous thrombosis correlate with development of recurrent thromboembolic events within the following 3–6 years. One of the main limitations of the study was that one could not determine the degree of risk of a thromboembolic event for a patient with elevated tPA [94].

### 17. Plasminogen activator inhibitor (PAI)

PAI- type 1 (PAI-1) inhibits tissue plasminogen activator (tPA). High levels of PAI-1 may be associated with an increased risk of arterial thrombosis due to inhibition of fibrinolysis [95]. Overall, the data available on the relationship between PAI-1 and the risk of thrombosis is conflicting. In 1996, Schulman and Wiman studied over 900 Swedish patients over 6 months after their initial presentation with a thrombotic event and found that elevated PAI-1 levels correlated with the risk of recurrence [94]. Using a cutoff level of 300 U/mL, 18% of subjects with recurrence had a higher level compared with 12% at a lower level (*P *= .045). In 2001, Crowther et al performed a prospective cohort study of 303 Canadian patients with a first episode of venous thromboembolism [96]. They conducted fibrinolytic testing and followed the patients for up to 3 years for recurrence. There was no clear difference in PAI-1 plasma levels in patients who did, or did not, suffer recurrence. There was no observed difference in levels between subjects who had idiopathic thrombosis compared to those with thrombosis in relation to another risk factor.

### 18. Thrombomodulin gene defect

Thrombomodulin is a major component of the protein C anticoagulant pathway. The protein C anticoagulant complex consists of thrombin (factor IIa) as the enzyme, thrombomodulin as the cofactor, and protein C as the substrate. As clot formation progresses, thrombin binds to thrombomodulin, an integral membrane protein on the endothelial cell surface [97]. Binding of thrombin to thrombomodulin induces a conformational change in thrombin, which changes its substrate specificity such that it acquires the ability to activate protein C and no longer promotes platelet activation or the cleavage of fibrinogen. A gene defect in the production of thrombomodulin will also affect the protein C anticoagulant pathway and will predispose an individual to thrombosis. However, biochemical detection of thrombomodulin defects is hampered by the location of the protein in the endothelial cell. The first mutation of thrombomodulin was diagnosed by Ohlin et al in 1995 in a 45 year-old male presenting with pulmonary embolism. Both the man and his healthy 21 year-old son were found to carry a heterozygous thrombomodulin gene substitution of G1456 to T [98]. Further investigations by Ohlin et al have found 8 heterozygous point mutations in 300 Swedish patients with thromboembolic disease [99]. The authors were able to show a statistically significant difference in allelic frequency between healthy and affected patients (p < 0.004) despite the small sample size.

### 19. Heparin Cofactor II

Heparin cofactor II (HC II) is a specific inhibitor of thrombin in the presence of heparin or dermatan sulphate. Studies in mice suggest an increased likelihood that HCII might inhibit thrombosis following arterial injury. In 1987, Bertina et al studied 277 Dutch patients with a history of unexplained venous thrombosis and identified three patients with a HC II below the lower limit of the normal range (60%) [100]. Family studies demonstrated hereditary HC II deficiency in two of the three cases. Among the 9 heterozygotes for HC II deficiency only one patient had a well-documented history of unexplained thrombosis. This and other studies suggest that although there have been reports on families in which a heterozygous HC II deficiency is associated with thromboembolic events, heterozygous HC II deficiency is as prevalent among healthy subjects as it is among patients with deep venous thrombosis [101]. It is, therefore, not yet clear whether HC II is, or is not, a thrombotic risk factor. Villa et al described a Spanish patient in 1999 who was found to be homozygous for HC II deficiency after suffering several thrombotic events [101]. Study of the patient's family showed that both the patient and her sister had homozygous HC II deficiency and that 12 of the 27 family members had heterozygous HC II deficiency. The patient was also found to have mild AT deficiency. Interestingly, all the 12 family members who were heterozygous for the deficiency, and the patient's sister, who was homozygous for HC II deficiency, were found to be healthy. The authors suggested that HC II deficiency may not play a limited in-vivo role as a thrombotic risk factor unless associated with AT deficiency or another congenital thrombotic risk factor.

### 20. Histidine-rich glycoprotein deficiency

Histidine-rich glycoprotein (HRG) is a single-chain glycoprotein. Platelets store HRG in the α-granules and secrete it upon thrombin activation. HRG has been shown to interact with plasma proteins involved in blood coagulation and fibrinolysis, however, the physiologic significance of molecular interactions is unclear [102]. Histidine-rich glycoprotein deficiency related to thrombophilia was first described in 1993 in a 43-year-old otherwise healthy Japanese woman who suffered from right transverse sinus thrombosis during oral contraceptive treatment [103]. Over the 6 months after stopping the drug, her plasma activities of antithrombin, protein C, protein S, heparin cofactor II, plasminogen and plasminogen activator inhibitor were normal, but her plasma histidine-rich glycoprotein (HRG) level was only 21% of the normal level of 109.5 +/- 51.5% (mean +/- 2 SD). Low levels of plasma HRG (20% to 35% of normal) were also found in her aunt, uncle and two daughters. Further analysis of the patient by Shigekiyo et al in 1998 demonstrated that a single missense mutation (nucleotide G429 to A in exon 3) leading to a mutation of Gly85 to Glu in the HRG molecule is a genetic cause for the first case of congenital HRG deficiency. At this time, further studies need to be carried out to determine the exact mechanism of HRG deficiency in thrombosis and the risk associated with individuals who are HRG deficient.

## Combined deficiencies

The varying degrees to which family members carry a certain defect has raised the possibility of individuals carrying more than 1 defect being more susceptible to thromboembolic events. In 1998, Mustafa et al suggested that there appeared to be an increased incidence of a second thrombophilic defect, particularly factor V Leiden, among patients who presented with thrombosis and were primarily found to have deficiencies of protein S or protein C [104]. In a study in Austria, factor V Leiden was present in four of fourteen patients with protein S deficiency and six of fifteen with protein C deficiency [104]. Carriers of two defects seem to be at a higher risk for thrombosis than their relatives with a single defect. In 1995, Koelman et al showed that APC-R was an additional risk factor for Dutch patients with protein C deficiency[105]. They found that 73% of the family members who had factor V Leiden combined with the protein C deficiency experienced a thromboembolic event, compared with 31% and 13% of carriers with a single defect of protein C deficiency or factor V respectively. In a retrospective case-control study in 2000, Gandrille studied 113 French individuals with known protein C deficiency and carried out genetic tests to determine presence of factor V Arg-Gln mutation [47]. 14% of those with protein C deficiency were found to have factor V mutation compared to 1% of the normal individuals. This and other studies have demonstrated that the simultaneous occurrence of hereditary thrombophilias and prothrombotic polymorphisms can substantially increase the risk of thromboembolic events in patients. In Germany, Boven et al studied 128 families in 1996 with AT deficiency and found that an additional factor V defect increased the likelihood of having a thromboembolic event at a younger age [106]. In individuals with both defects, the percentage of symptomatic individuals with both abnormalities was 80%. In 1999, Salomon et al published a casecontrol study of 162 Israeli patients with at least one episode of venous thromboembolic disease compared with 336 healthy controls [107]. Two or more polymorphisms were detected in 27 of 162 patients (16.7%) and in 3 of 336 controls (0.9%). The authors of the study demonstrated an odds ratio of 58.6 (confidence interval [CI], 22.1 to 155.2) for joint occurrence of factor V and prothrombin gene polymorphisms, of 35.0 (CI, 14.5 to 84.7) for factor V and MTHFR polymorphisms, and of 7.7 (CI, 3.0 to 19.6) for factor II and MTHFR polymorphisms. They concluded that, in the study population, the presence of greater than one of the prothrombotic polymorphisms was associated with a substantial risk of venous thromboembolism.

In 2001, Emmerich et al conducted a pooled analysis of 8 case control studies looking at 2310 cases and 3204 controls to estimate the risk of thrombosis in double heterozygotes for factor V Leiden and the prothrombin G20210A mutations [108]. Among the population studied, 12% percent of those heterozygous for factor V Leiden were also heterozygous for factor II G20210A, while 23% of patients heterozygous for factor II G20210A were also heterozygous for factor V Leiden. The data showed an odds ratio for venous thrombosis in double heterozygotes of 20.0 (11.1–36.1). This was much higher than the odds ratios for thromboembolism of 4.9 (95% CI; 4.1–5.9) for the factor V Leiden and 3.8 (3.0–4.9) for the factor II G20210A mutation as single defects [108].

## Conclusion

The risk of thrombosis in the pediatric population is less than that in adults. However, thromboembolic disease represents a significant source of mortality and morbidity, and venous thromboembolism continues to present a challenge to clinicians. It is important to note that there are various settings such as surgery, flights etc. that can lead to thrombosis in otherwise healthy individuals who have thrombophilic defects. In recent years, there have been major advances in our understanding of congenital defects that predispose to thrombosis. This has led to a more complete understanding of the disease processes, as well as recommendations for appropriate screening, detection, diagnosis and treatment.

Current treatment for thrombophilias involves both prophylaxis with low-molecular-weight heparin and treatment involving heparin, warfarin or purified factor concentrate. The presence of an inherited thrombophilia should not alter the intensity of anticoagulant therapy, given that antithrombin, protein C, or protein S deficiency, factor V Leiden, and the prothrombin G20210A mutation are not unusually anticoagulant resistant. However, they can increase the optimal treatment duration after a first thromboembolic event [109].

Clinical trials on treatment are essential since they will provide physicians with the information to determine whether or how they should modify their clinical practice. Correctly identifying hereditary risk factors, together with appropriate genetic evaluation and counseling, will allow the informed patient and physician to work together for effective management of thrombophilia and prevention of subsequent thrombotic events.

## Competing interests

The author(s) declare that there are no competing interests.

## Authors' contributions

SK and JDD both designed and conceived of the topic for review. SK researched the literature and wrote the paper. JDD reviewed and edited the paper.
